# Commentary: Commentary: Synergistic treatment of sodium propionate and Sishen Pill for diarrhea mice with kidney-yang deficiency syndrome

**DOI:** 10.3389/fcimb.2025.1650698

**Published:** 2025-08-13

**Authors:** Junxi Shen, Zhoujin Tan

**Affiliations:** School of Traditional Chinese Medicine, Hunan University of Chinese Medicine, Changsha, Hunan, China

**Keywords:** kidney-yang deficiency syndrome, diarrhea, gut-kidney axis, microbial metabolites, Sishen Pill, intestinal microbiota, dysbiosis

## 1 Introduction

Sishen Pill is endorsed as the primary therapeutic option for treating diarrhea with kidney-yang deficiency syndrome in the “Consensus of traditional Chinese medicine (TCM) Experts on the Diagnosis and Treatment of Diarrhea.” Recently, Guo et al. published an article titled “Synergistic treatment of sodium propionate and Sishen Pill for diarrhea mice with kidney-yang deficiency syndrome” in *Frontiers in Cellular and Infection Microbiology*. This study found that a combined regimen consisting of 75% Sishen Pill + 60 mg/kg sodium propionate could regulate the intestinal microecology and ameliorate the symptoms in mice with diarrhea with kidney-yang deficiency syndrome. Although Zhao et al. (2025) have carried out discussions from three dimensions, namely causality, pharmacokinetics, and the neuroendocrine dimension, we believe that there are still deficiencies in the research approaches concerning aspects such as causality. Therefore, we deem it necessary to engage in more in-depth reflections on these dimensions. This will be more conducive to facilitating the in-depth advancement of research in TCM and microecology.

## 2 General comments

To uncover the pathomechanisms of diarrhea with kidney-yang deficiency syndrome and Sishen Pill therapeutic actions, our team established a mouse model replicating the syndrome via experiments (Li et al., 2023; Zhu et al., 2022). Our findings indicate that the intestinal microbiota balance in mice with diarrhea with kidney-yang deficiency syndrome is disrupted (Zhou et al., 2024). Sishen Pill is capable of inducing alterations in the structure and function of the intestinal microbiota in mice with diarrhea with kidney-yang deficiency syndrome, thereby promoting the production of short-chain fatty acids such as propionic acid and butyric acid (Li et al., 2024).

Guo et al. innovatively adopted a combined therapeutic strategy, utilizing Sishen Pill in conjunction with sodium propionate, to treat diarrhea with kidney-yang deficiency syndrome. Their research revealed that a treatment regimen comprising 75% Sishen Pill + 60 mg/kg sodium propionate exerted a notably positive regulatory influence on intestinal microecology, consequently alleviating the associated symptoms (Guo et al., 2025a). Recently, Zhao et al. published a commentary on this research (Zhao et al., 2025). We would like to offer further commentary on the aforementioned commentary.

### 2.1 The causal relationship between microbial changes and diarrhea alleviation is unclear

Zhao et al. proposed utilizing fecal microbiota transplantation (FMT) studies in germ-free models to verify causality (Zhao et al., 2025). However, The absence of microbiota in germ-free animal models leads to various structural and functional abnormalities, including alterations in intestinal villus morphology, reduced expression of antimicrobial peptides at mucosal barrier sites, and so on. Importantly, under germ-free conditions, there is a reduced number of innate immune cells in the intestine, and the immune system is underdeveloped. The immune deficiencies in germ-free models may interfere with host-microbiota interactions (Thomson et al., 2022; Jans and Vereecke et al., 2025). Moreover, the complexity and variability of FMT make it difficult to accurately elucidate its functions, and the standard definition of intestinal microbiota from healthy donors remains rather ambiguous (Yadegar et al., 2024; Yi et al., 2024). Therefore, we believe that focusing on the intestinal microecological mechanisms exhibited by the organism under normal conditions may better align with the characteristics of microorganisms.

### 2.2 Lack of pharmacokinetic studies on Sishen Pill and sodium propionate

Zhao et al. suggested that Caco-2 transwell assays and hepatic microsomal stability testing could provide in-depth insights into the absorption and metabolism processes of drugs *in vivo*, thereby offering a scientific basis for optimizing combined drug regimens (Zhao et al., 2025). Actively conducting pharmacokinetic experiments is highly necessary for drug development. This will provide insights into drug - drug interactions, individual differences in drug efficacy, and drug target prediction (Lai et al., 2022). Currently, validating the efficacy and safety of this combo therapy in diverse animal/preclinical models and gathering more foundational data are vital. In future studies, comprehensive *in vitro*/*in vivo* pharmacokinetic studies are feasible on Sishen Pill. These studies encompass assessments such as artificial membrane permeability, recombinant enzyme metabolism, and 3D cell culture studies. For instance, the use of hepatic spheroid co-culture models enables highly sensitive prediction of drug-induced liver injury risk (Wang et al., 2021).

### 2.3 The biological connotation of kidney-yang deficiency syndrome

Zhao et al. proposed the need to focus on the neuroendocrine dimension of kidney-yang deficiency syndrome, particularly the regulatory role of the hypothalamic-pituitary-adrenal (HPA) axis (Zhao et al., 2025). Kidney - yang deficiency syndrome is closely related to dysfunctions of HPA axis hormones and is characterized by an imbalance in the mutual control among pituitary - target gland axis hormones (Ayu et al., 2020). They also mentioned that the modern understanding of the TCM concept of “kidney governing water metabolism” would offer assistance in the treatment of diarrhea. We also believe that this will contribute to the modern understanding of TCM theory. For instance, the pivotal roles of energy metabolism or water metabolism balance in diarrhea with kidney-yang deficiency syndrome. α-Enolase (ENOA), a highly conserved cytoplasmic glycolytic enzyme, is downregulated in the colon mucosa of irritable bowel syndrome (IBS) patients, disrupting glycolysis. These alterations may reduce substrates for the tricarboxylic acid (TCA) cycle and oxidative phosphorylation, affecting ATP levels and related reactions. The resulting energy deficiency impairs colonocyte function, leading to intestinal disorders such as diarrhea (Zhang et al., 2019; Chey et al., 2015). The intestinal microbiota and its metabolites, functioning as mediating factors, may regulate water metabolism balance through three mechanisms, thereby influencing the onset and progression of diarrhea: aquaporins and ion channels, the intestinal mucosal barrier, and the renin-angiotensin-aldosterone system (Yu et al., 2025). Future research based on the aforementioned content will help unveil the pathological mechanism of diarrhea with kidney-yang deficiency syndrome.

## 3 Discussion

Zhao et al. conducted a holistic analysis of the research on the “synergistic treatment of sodium propionate and Sishen Pill for diarrhea in mice with kidney-yang deficiency syndrome”. They not only acknowledged the value of this study but also pointed out the limitations and ambiguities in its underlying mechanisms, while proposing numerous key research directions. The latest research findings from Guo et al. demonstrate that a combination of 75% Sishen Pill and 60 mg/kg sodium propionate can treat diarrhea with kidney-yang deficiency syndrome by regulating the intestinal microbiota, enhancing intestinal immune function, reducing IL-6 levels, and alleviating inflammation (Guo et al., 2025b). This will assist researchers in gaining a clearer understanding of the therapeutic potential of combining metabolic regulation with TCM in restoring intestinal homeostasis.

Intestinal microbiota, by virtue of its crucial regulatory role in human physiological and pathological processes, has become a bridge connecting TCM theory with modern science. We hope that future researchers will give full consideration to the following aspects: 1. Greater emphasis should be placed on the intrinsic characteristics of microorganisms in intestinal microbiota research; 2. Proactively conduct pharmacokinetic studies from multiple perspectives; 3. Pay attention to the biological connotations of TCM syndromes and clarify their essence. By refining research content through these directions, we aim to explore the therapeutic mechanisms of Sishen Pill and promote the modernization of TCM.
